# Aging and Death in E. coli

**DOI:** 10.1371/journal.pbio.0030058

**Published:** 2005-02-01

As human beings, aging and death are an inevitable part of our lives. As we pass through each decade, the concrete signs of aging—greying hair, aches and pains, the gradual failure of one organ system after another—and the realization that we are mortal increasingly occupies our thoughts.

All other multicellular animals and plants also show clear signs of aging, as do some single-celled organisms. In the yeast Saccharomyces cerevisiae (baker's yeast), for example, the function of individual cells gradually declines with time, and each yeast cell has a finite life span. In organisms like this, it has been proposed that reproduction by asymmetric division is a prerequisite for aging. In other words, for a unicellular organism to age, when it divides, it must give rise to a “parent” cell and a smaller offspring cell (as in yeast), which then has to go through a juvenile phase of growth or differentiation before it divides. At each cell division, the parent cell becomes older until it reaches its natural life span and dies.[Fig pbio-0030058-g001]

**Figure pbio-0030058-g001:**
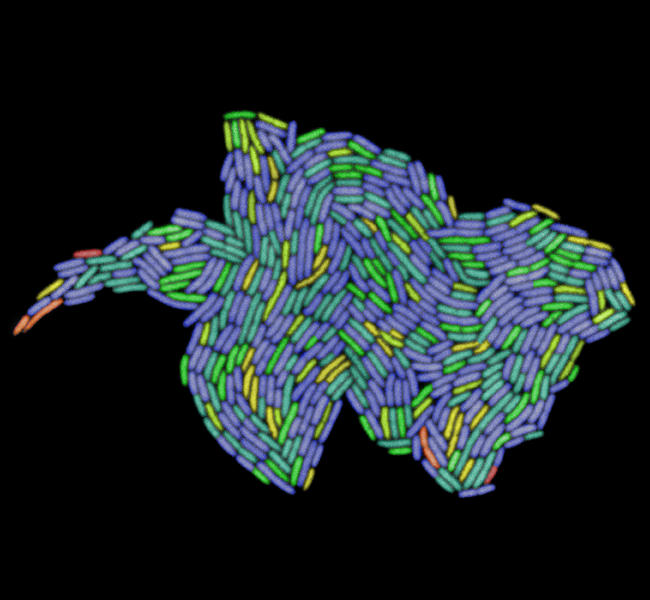
A growing microcolony of E. coli

But what about organisms that produce two apparently identical cells when they divide? Do such organisms age? The assumption has been for some years that cells that divide symmetrically do not age and are functionally immortal. Eric Stewart and colleagues have now tested this idea by analyzing repeated cycles of reproduction in Escherichia coli, a bacteria that reproduces without a juvenile phase and with an apparently symmetric division.

E. coli is a rod-shaped organism that reproduces by dividing in the middle. Each resultant cell inherits an old end or pole and a new pole, which is made during the division. The new and the old pole contain slightly different components, so although they look the same, they are physiologically asymmetrical. At the next division, one cell inherits the old pole again (plus a brand new pole), while the other cell inherits, a not-quite-so-old pole and a new pole. Thus, Stewart and co-workers reasoned, an age in divisions can be assigned to each pole and hence to each cell.

The researchers used automated time-lapse microscopy to follow all the cell divisions in 94 colonies, each grown from a single fluorescently labeled E. coli cell. In all, the researchers built up a lineage for 35,049 cells in terms of which pole—old or new—each cell had inherited at each division during its history. They found that the cells inheriting old poles had a reduced growth rate, decreased rate of offspring formation, and increased risk of dying compared with the cells inheriting new poles. Thus, although the cells produced when E. coli divide look identical, they are functionally asymmetric, and the “old pole” cell is effectively an aging parent repeatedly producing rejuvenated offspring.

Stewart and his colleagues conclude that no life strategy is immune to the effects of aging and suggest that this may be because immortality is too costly or is mechanistically impossible. This may be bad news for people who had hoped that advances in science might eventually lead to human immortality. Nevertheless, E. coli should now provide an excellent genetic platform for the study of the fundamental mechanisms of cellular aging and so could provide information that might ameliorate some of the unpleasantness of the human aging process.

